# Severe anemia and melena caused by pyeloduodenal fistula due to renal stone-associated squamous cell carcinoma

**Published:** 2014

**Authors:** Jian Hui Wu, Yong Xu, Zi Qiang Xu, Kuo Yang, Shi Qiang Yang, Hong Shun Ma

**Affiliations:** 1 Jian Hui Wu, Department of Urology, Second Hospital of Tianjin Medical University, Tianjin Institute of Urology, Tianjin First Center Hospital, Tianjin 300211, China.; 2 Yong Xu, Department of Urology, Second Hospital of Tianjin Medical University,Tianjin Institute of Urology, Tianjin 300211, China.; 3 Zi Qiang Xu, Department of Urology, Tianjin First Center Hospital, Tianjin 300192, China.; 4 Kuo Yang, Department of Urology, Second Hospital of Tianjin Medical University,Tianjin Institute of Urology, Tianjin 300211, China.; 5 Shi Qiang Yang, Department of Urology, Tianjin First Center Hospital, Tianjin 300192, China.; 6 Hong Shun Ma, Department of Urology, Tianjin First Center Hospital, Tianjin 300192, China.

**Keywords:** Calculus, Digestive tract, Pyeloduodenal fistula, Squamous cell carcinoma, Urinary tract

## Abstract

Pyeloduodenal fistula is a rare condition and its association with malignancy is even rarer. Herein we report the case of a 66-year-old man who was admitted to the hospital with a three-month history of intermittent melena and a more than 20-year history of right-side renal stones. Computed tomography showed a heterogeneous right renal mass with a staghorn stone that had invaded the duodenum and caused an internal fistula. An upper gastrointestinal series showed pyeloduodenal fistula. The patient underwent an exploratory operation and a biopsy was taken at the peripelvic region. Pathological examination verified the existence of squamous cell carcinoma. To our knowledge, this is the first case of pyeloduodenal fistula associated with renal stones, squamous cell carcinoma and upper urinary tract calculus presenting on melena. We report on the features of this rare entity but also review and summarize the etiology, diagnosis and treatment options that can be extrapolated from the existing literature.

## INTRODUCTION

Pyeloduodenal fistula is a rare condition and its association with malignancy is even rarer. We report a case of pyeloduodenal fistula associated with squamous cell carcinoma and upper urinary tract calculi presenting with melena. This combination of symptoms and conditions has not been described in previous reports.

## CASE REPORT

**Fig.1:**
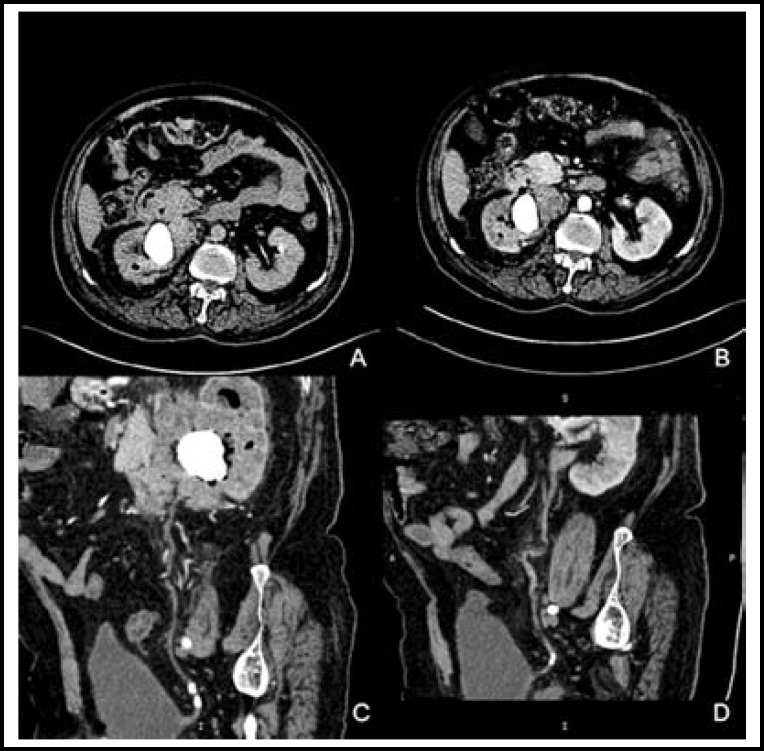
Computed tomography.

**Fig.2:**
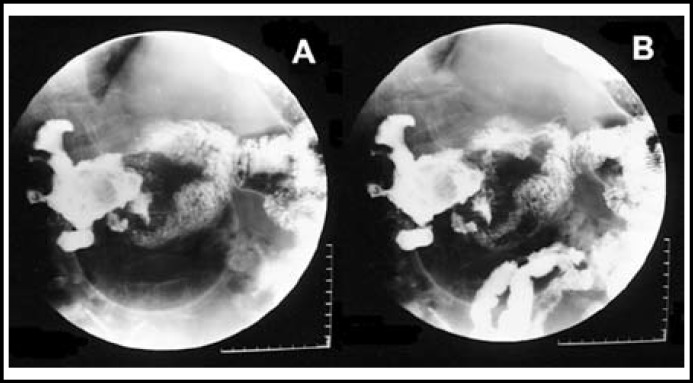
Upper gastrointestinal series.

A 66-year-old man was admitted to the hospital with a three-month history of intermittent melena, nausea, malaise, and upper abdominal pain that worsened significantly two weeks prior to his admission. The patient had more than a 20-year history of right renal stones. He also reported weight loss of 15 kg during the prior six months. Physical examination revealed a pale, frail old man with a temperature of 36.8˚C, and a soft abdomen with moderate tenderness in the right upper quadrant. Ultrasonography showed right renal parenchymal damage and a staghorn stone. Abdominal computed tomography (CT) showed a heterogeneous renal mass containing a staghorn stone that had invaded the duodenum (an internal fistula), air localized in the right renal pelvis, and a thrombosed inferior vena cava. Further, computed tomography urography (CTU) revealed bilateral ureteric calculi (Fig.1). An upper gastrointestinal series showed that contrast filled the small intestine and simultaneously overflowed into the right, dilated renal pelvis at the second part of the duodenum (Fig.2). Single photon emission computed tomography (SPECT) reported the total glomerular filtration rate as 55.6 mL/min per 1.73 m^2^ and the split renal functional ratio as 84.9% and 15.1% for the left and right kidneys, respectively. Urine culture subsequently grew *Escherichia coli* (> 10^5^ CFU).

Initial treatment of the patient consisted of blood transfusion, intravenous sulperazone (a combination of cefoperazone and sulbactam), parenteral nutrition support, and analgesic therapy. Reassuringly, his vital signs became stable after one week and he resumed a normal diet, but the right flank pain worsened. After consultation with appropriate experts, we suggested the patient have an exploratory surgery to provide a therapeutic opportunity with which to place a percutaneous tube to drain the bladder and bowel. We also analyzed other known prognostic variables to try to explain the poor prognosis. Two weeks later, the patient underwent the exploratory operation. A retroperitoneal mass was found arising from the right pelvis. The second part of the duodenum and the hepatic flexure of the colon were both fixed with the mass. The mass also wrapped around the renal artery and inferior vena cava, invaded the psoas major muscle and was fixed. The fistula and inferior vena cava were undetectable. Therefore, when we concluded from the exploratory surgery that there was no chance of en bloc excision, and we gave up on a surgical solution. Moreover, the surgeons could not disregard the effect of palliative surgery involving multiple systems and the economic situation of the patient’s family. However, a biopsy was taken at the peripelvis. The pathologic examination revealed squamous cell carcinoma. Due to economic circumstances, sociocultural factors and local customs, the patient returned to home hospice and unfortunately died less than five months later.

## DISCUSSION

There are almost 100 cases of pyeloduodenal fistulas that have been reported since the first case was described by Campaignac in 1839.^1^^,^^2^ Only eight cases of pyeloduodenal fistulas were due to upper tract malignancy (Table-I).^2^^-^^9^ Most pyeloduodenal fistulas occur because of a chronic renal inflammatory disease, such as pyonephrosis, perinephritis, renal calculi, xanthogranulomatous pyelonephritis, or tuberculosis. Less commonly, they are of a duodenal origin, such as a duodenal ulcer or hydatid cyst of the kidney or malignancy of the upper digestive tract.^3^ The etiology of traumatic pyeloduodenal fistula had been reported as due to falls, crush injuries, ureteral catheterizations, open surgeries, foreign bodies, percutaneous surgeries and gunshot wounds.^10^ The anatomic proximity between the right renal pelvis and the second portion of the duodenum is an important factor. Calculi, obstruction, chronic inflammation and infection have generally also been considered pivotal causes.

and (B): Transverse views showing the right renal mass, kidney calculus, and air localized in the right renal pelvis with part of the ascending colon; the inferior vena cava was encompassed and thrombosed. (C) and (D): In sagittal reconstructed views showing bilateral distal ureteral calculi.

**(A)** **:** **Oral contrast passing the duodenum and the corroded right renal pelvis. (B):** **Oral contrast entering the small intestine.**

**Table-I:** Pyeloduodenal fistula due to upper urinary tract malignancy

*Reference*	*Report*	*Age*	*Sex*	*Etiology*	*Result*
Jones et al.^*^	1953	65	no	Epidermoid carcinoma, calculus	no
Cohen et al.	1966	54	M	Pyelonephritis, perinephric abscess, renal papillary carcinoma	Satisfactory
Tsuchiaya et al.	2001	62	F	Adenocarcinoma, stones	Satisfactory
Chen et al.	2002	73	F	Renal transitional cell carcinoma	Death
Poon et al.	2003	75	M	Squamous cell carcinoma, stones	Death
Hernandez et al.	2007	71	F	Urothrelial carcinoma	Death
Chung et al.	2008	74	M	Squamous cell carcinoma, stone	Satisfactory
Ruiz Plazas et al.^**^	2008	49	M	Renal carcinoma, calculus	no

F = female; M = male.

* This case comes from the report by Cohen et al.

** This case was reported in Spanish. ”no” means there is no described material.

Presenting symptoms that have been reported include a variety of urinary tract, gastrointestinal and constitutional symptoms as a result of involvement of both the digestive and urinary systems. In the review by McEwan,^1^ it was pointed out that an insidious and progressive asymptomatic pyelonephritis was an attendant clinical condition, but the gastrointestinal symptoms should be considered an important clue for diagnosis. There are several reviews that have reported that diarrhea, nausea, vomiting, epigastric pain, dyspepsia, general malaise and weight loss are common symptoms, but there was only one reported case of presentation with melena.^6^ Gastrointestinal hemorrhage presents as severe bleeding with hematemesis, hematochezia, and hypotension, or as gradual bleeding with melena. Our case initially presented with severe, chronic anemia due to melena. Another rare symptom is hyperchloremic metabolic acidosis due to urine reabsorption by the bowel, Only rare cases with renal failure present with hyperchloremic metabolic acidosis because of a poorly functioning right kidney and minimal urine entering the gastrointestinal tract.

Many case reports and reviews have advocated retrograde or antegrade pyelogram as the proper treatment choice for pyeloduodenal fistulae. Intravenous urography infrequently demonstrates existence of a fistula due to poor function of the affected kidney.^3^ In fact, a large number of these fistulae have been demonstrated radiographically, with oral contrast examinations such as CT and upper gastrointestinal series.

Nephrectomy and primary closure of the duodenum are the traditional treatments for most non-traumatic pyeloduodenal fistulae. Patients with a non-traumatic pyeloduodenal fistula usually have minimal function. In contrast to such cases, every effort is made in patients with a traumatic pyeloduodenal fistula to salvage the functioning kidney. Effort is also made to patch the duodenum with a tongue omentum and nasogastric tube. Ginsberg et al.^10^ suggested that conservative therapy should be the first line of treatment for patients who have had percutaneous stone surgery. Recent advances in technology for percutaneous renal surgery have led to reports of several successful experiences with conservative treatment, providing encouragement that new and successful treatments can be developed. However, only four of eight cases associated with malignancy and described in published studies had palliative operations and no additional administration of chemotherapy. More than 50% of patients with malignant pyeloduodenal fistula died from post-operative complications, such as sepsis, respiratory failure and multiple organ failure.^2^^-^^9^ The rare case presented in this report illustrates that renal stones associated with squamous cell carcinoma can present with pyeloduodenal fistula and melena. It also demonstrates that pyeloduodenal fistula due to malignancy can be managed by radical or palliative surgery, although in the current case, the complicated conditions of the patient were difficult to effectively manage surgically.
